# Everybody's Page

**Published:** 1890-06-21

**Authors:** 


### EVERYBODY'S PAGE.

To most people the sight of bent-wood furniture is familiar, and now it seems that bent-wood cart-wheels are to come into use. The advantage over those made in the usual way is not only that the wood can be rendered very pliable, but that the manufacture is also cheap.

This is a story of a lady who asked a dirty-looking female tramp, who begged a copper, why she did not work: "I am working, lady." "Oh are you; how?" "I'm the travelling advertisement of a soap firm; I'm the 'before using' card, and my friend, who's just round the corner, is the 'after using.'"

A cork rope is one of the latest inventions; it is made of small corks, placed end to end, and the whole covered with a braiding of cotton twine; over this is a coarser braiding in heavy strands. According to the inventor, a one-inch-thick rope will stand a strain of a thousand pounds. A really-successful floating rope at a life-saving station would be invaluable.

This year is interesting as being the Tercentenary of Jansen's microscope, believed to be the first microscope ever made, though the magnifying power of lenses must have been known for ages. Jansen, however, combined lenses, and the result was a very imperfect instrument, which coloured and distorted objects, and was looked upon at first more as a plaything than anything which would prove an indispensable to scientific progress.

According to the *Cologne Gazette*, the German doctor Alanus, notorious for his advocacy of vegetarian diet, has become a pervert and has returned to a mixed diet of flesh and vegetable food. He owns that one brutal fact has overturned all his theoretical preaching, and that after having been a vegetarian for a number of years without feeling any bad or good effect he suddenly observed that his arteries began to show signs of degeneration. Being still a young man this morbid symptom was not due to old age, therefore Dr. Alanus puts it down to vegetarian diet.

Dr. Coupland Taylor, in his work entitled "Wanderings in Search of Health," declaims against the practice of sending phthisical patients on voyages under conditions which are favourable to the propagation of the disease. It is quite true that no one has a right to endeavour to save himself at the cost of injury to the health of his neighbours, but Dr. Taylor's advocacy of the necessity of largely increasing the number of single-berthed cabins in vessels, is hardly likely to meet with the approval of shipowners. Unfortunately room is limited on board ship.

The question of smoke-abatement and the adoption of smoke-preventing appliances is one that has been before the public now for so long, that we almost despair of anything practical being done. Lord Derby, we think, hit the mark on Thursday last, when he said that the real difficulty to be encountered is indifference, and indifference curiously enough on the part of the very people who from their sufferings should be interested in abating the smoke nuisance. Every inhabitant of a town is concerned in the diminution of smoke and its accompanying dirt, and obtaining the purest possible air.

The inhabitants of Sheffield must be truly thankful that the origin of the lead-poisoning, which has had such disastrous effect upon their health, has been traced to its source. A majority of the population of the town and surrounding district appears to have suffered from the most intense forms of the poisoning.

While some lovers of books shut their treasures up in tight cases, hoping thereby to protect them from the ravages of time, others declare that pure dry air is an essential to the long life of a book just as it is to a human being. Perhaps one of the least expensive and best methods of protection is to hang a thin curtain over the shelves, and thus keep out the dust.

It is proposed by the Northern France Butter Syndicate that a law shall be passed compelling adulterators of butter to colour their compounds with some colouring matter other than butter colour. Normandy butter is fast losing its name for purity; and this sounds a useful suggestion, for few of us would care to eat butter of various hues, and detection of margarine would then be a simple matter.

If any further evidence is needed to show the desirability of closing all cemeteries in large towns and resorting to cremation, Paddington cemetery affords plenty. Why the stagnant pools of water caused by the overflow from it during any heavy rain-fall have not resulted in a widespread outbreak of malignant fever in its immediate neighbourhood is due to extraordinary good fortune. It must be a relief to the inhabitants to know that a surface drainage has recently been put in.

The influence of European civilization in India is, as must inevitably be the case, gradually weakening many orthodoxies which do not commend themselves to western ideas. Soap, a luxury almost unknown hitherto among natives of India, is at last claiming some attention from them. Soaps made of animal fat are, of course, repugnant to the orthodox Hindu, but now that he can get vegetable soaps he can be both orthodox and clean, and to do him justice he appears to have no objection to being so.

In the canton of Geneva a sanitary inspection of infant and primary schools is made at least twice during the year, once in January, and again after the summer holidays. How strict this examination is, is shown by the fact that each child is overhauled individually. Every child who shows symptoms of any contagious disease has to be sent home at once, and is not allowed to return to school without presenting a doctor's certificate to the effect that his or her return will have no bad consequences for the rest of the school.

The Chinese, who reject scornfully nearly every application of Western Medical Science, are, according to the Governor of Hong Kong, firm believers in the advantage gained from vaccination, and submit to the ordeal with a cheerfulness and philosophy which are characteristic of this wily oriental. Protection by vaccination is especially required in Hong Kong, owing, as Sir William Des Vœux points out, to the frequency with which small-pox is introduced by steamers coming from all parts of the world, and to its fatal prevalence when it has once obtained a footing.

